# Microbiota of wild-caught Red Snapper *Lutjanus campechanus*

**DOI:** 10.1186/s12866-016-0864-7

**Published:** 2016-10-21

**Authors:** Andrea M. Tarnecki, William F. Patterson, Covadonga R. Arias

**Affiliations:** 1 Mote Marine Laboratory, 1600 Ken Thompson Parkway, Sarasota, FL 34236 USA; 2 Department of Marine Sciences, University of South Alabama, Life Sciences Building Room 25, Mobile, AL 36688 USA; 3 Auburn University, School of Fisheries, Aquaculture, and Aquatic Sciences, 203 Swingle Hall, Auburn, AL 36849 USA

**Keywords:** Microbiota, *Lutjanus campechanus*, Aquaculture, Pyrosequencing

## Abstract

**Background:**

The microbiota plays an essential role in host health, particularly through competition with opportunistic pathogens. Changes in total bacterial load and microbiota structure can indicate early stages of disease, and information on the composition of bacterial communities is essential to understanding fish health. Although Red Snapper (*Lutjanus campechanus*) is an economically important species in recreational fisheries and a primary aquaculture candidate, no information is available on the microbial communities of this species. The aim of this study was to survey the microbiota of apparently healthy, wild-caught Red Snapper from the Gulf of Mexico. Sampled Red Snapper showed no physical signs of disease. Tissues that are either primary entry routes for pathogens (feces, gill) or essential to disease diagnosis (blood) were sampled. Bacteria were enumerated using culture-based techniques and characterized by pyrosequencing.

**Results:**

Aerobic counts of feces and gill samples were 10^7^ and 10^4^ CFU g^-1^, respectively. All individuals had positive blood cultures with counts up to 23 CFU g^-1^. Gammaproteobacteria dominated the microbiota of all sample types, including the genera *Pseudoalteromonas* and *Photobacterium* in feces and *Pseudomonas* in blood and gill. Gill samples were also dominated by *Vibrio* while blood samples had high abundances of *Nevskia*. High variability in microbiota composition was observed between individuals, with percent differences in community composition ranging from 6 to 76 % in feces, 10 to 58 % in gill, and 52 to 64 % in blood.

**Conclusions:**

This study provides the first characterization of the microbiota of the economically significant Red Snapper via pyrosequencing. Its role in fish health highlights the importance of understanding microbiota composition for future work on disease prevention using microbial manipulation.

## Background

In 2012, aquaculture operations produced over 66 million tonnes of food fish worth nearly $140 billion US dollars, with total aquaculture production 14 times higher than that of 1980 [1]. It is estimated that by 2030, 63 % of the world’s food fish will be produced in aquaculture [2], as a vast majority of wild fish stocks are fully or overfished with no room for expansion of commercial fishing efforts [1]. However, disease remains a significant limitation to the growth of the aquaculture industry [3, 4] and is responsible for losses valued at billions of dollars each year [5]. Microbial communities, known as microbiota, play a large role in maintaining host health through increasing digestion efficiency and use of nutrients, boosting the immune system, and preventing attachment and proliferation of opportunistic pathogens [6, 7]. Interest in manipulation of the microbiota to take advantage of these benefits and to prevent disease in aquaculture has increased dramatically [4, 8, 9]. However in many fish species, the composition of the natural microbiota has not been characterized and as a result, the dominant bacterial players and their downstream influence on fish health are unclear.

Documenting the bacteria present in healthy individuals is an essential first step to understanding the impacts of microbial manipulation in aquaculture systems. As it pertains to disease resistance, the microbiota associated with gill and intestine are of particular concern as these are primary entry routes for opportunistic pathogens in fishes [10, 11]. The bacterial abundance and diversity at these sites can provide insight into the health status of individuals as abundance of opportunistic pathogens increases and bacterial diversity decreases during stress and times of disease [12, 13]. Monitoring blood is also important as bacterial septicemia or bacteremia is diagnosed when bacteria are recovered from internal organs such as liver and anterior kidney [14]. Often, Koch’s postulates are not performed and isolation of bacteria from a diseased fish is deemed sufficient for diagnosis [15]. However, the presence of bacteria from the blood and/or internal organs of apparently healthy individuals [16–26] challenges the notion that a positive blood culture is indicative of disease in fish.

Red Snapper *Lutjanus campechanus* (Poey, 1860) is an economically and ecologically significant reef fish species in the Gulf of Mexico, contributing to the $199 billion of sales impacts generated by recreational and commercial fisheries in the US [27]. The Red Snapper stock in the US waters of the Gulf of Mexico was estimated to be severely depleted by the 1980s; however, management strategies implemented by the Gulf of Mexico Fishery Management Council in mid 2000s are projected to recover the stock above its biomass threshold by 2032 [28]. The economic value of Red Snapper as a food and game fish as well as its wild stock status make this species a primary aquaculture candidate [29]. As a result, disease diagnosis in this species is relevant to the aquaculture industry. There is very little information on the microbial communities associated with Red Snapper including identification of potential pathogens for the species. In a previous study [24], we showed that apparently healthy Red Snapper harbored bacteria in their internal organs and identified bacteria associated with Red Snapper skin. However, we used culture-dependent methods which may underestimate bacterial diversity by 90–99 % [30]. No studies to date have investigated the microbiota of Red Snapper using culture-independent methods, including next-generation sequencing (NGS) technologies which allow for thorough detection and characterization of both dominant and rare members of the microbiota [31].

Due to the relevance of the microbiota in fish health and the interest in microbial manipulation to control diseases in aquaculture systems, this study aimed to thoroughly characterize microbiota associated with gill, intestine, and blood of wild, healthy Red Snapper, a primary aquaculture candidate. We used culture-based methods to determine bacterial load and NGS techniques to identify dominant bacterial taxa, providing essential information on healthy bacterial community structure for future investigations into microbiota functions, pathogen identification, and health monitoring in this economically significant fish species.

## Results

### Site conditions and fish sampled

Average environmental conditions at the sampled sites (Table 1) were as follows: depth – 28 m, salinity – 34 psu, temperature – 26 °C, DO – 6.3 mg L^−1^, fluorescence – 0.14 mg (m^3^)^−1^, turbidity – 89.6 %. A total of six male and four female Red Snapper were sampled with a mean weight of 1.3 ± 1.6 kg and mean total length of 434 ± 114 mm.

**Table 1:** Sampling locations, environmental conditions, and Red Snapper characteristics

		Site 1		Site 2		Site 3		Site 4		Site 5	
	Coordinates	30° 00' N		30° 09' N		30° 02' N		30° 02' N		30° 02' N	
		87° 42' W		87° 09' W		87° 39' W		87° 34' W		87° 34' W	
	Sampling date	26-Sep		30-Sep		17-Oct		13-Nov		13-Nov	
Environmental conditions	Depth (m)	30.9		26.5		26.9		27.9		28.5	
	Salinity (psu)	33.6		33.1		34.1		34.5		34.5	
	Temperature (°C)	28.6		28.8		27.2		22.7		22.7	
	Dissolved oxygen (mg L^−1^)	4.77		6.02		6.18		6.68		7.71	
	Fluorescence (mg (m^3^)^−1^)	0.134		0.135		0.089		0.187		0.178	
	Turbidity (% saturation)	88.2		91.5		90.1		89.4		88.7	
Fish collected	Snapper ID	1	2	3	4	5	6	7	8	9	10
	Sex	F	M	F	F	M	M	M	F	M	M
	Mass (kg)	1.13	0.56	0.83	6.1	0.46	0.46	1.52	0.64	0.74	0.86
	Length (mm)	440	405	405	752	335	338	487	389	395	395

Environmental conditions were recorded on a Seabird 19+ CTD. Two Red Snapper were caught from each site

### Aerobic heterotrophic counts

**Fig. 1:**
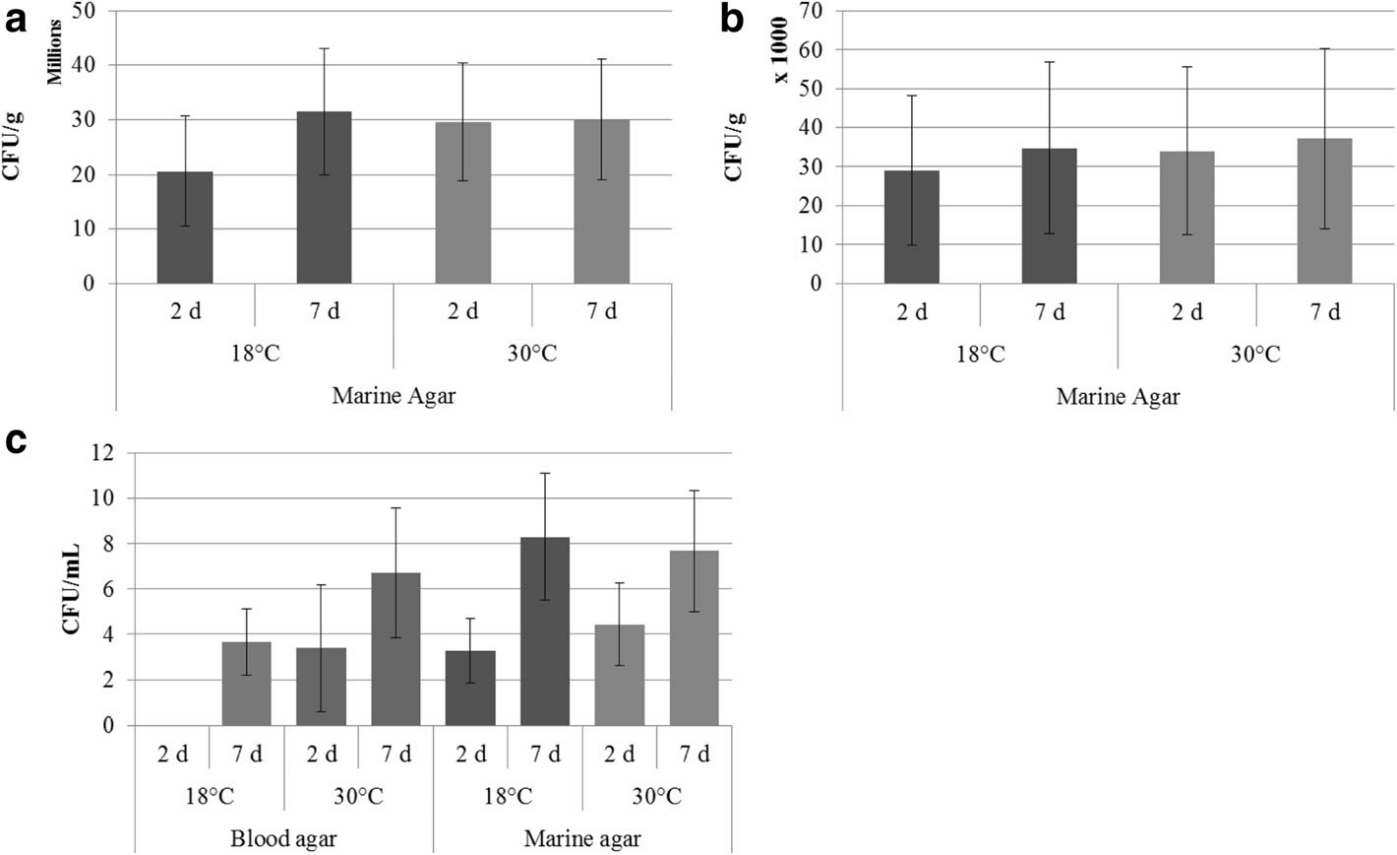
Average colony forming units (CFUs) ± standard error. **a** feces, **b** gill, and **c** blood sample types by day, temperature, and media

After 1 week incubation on MA at 30 °C, CFU g^−1^ of feces ranged from 5.33 × 10^4^ to 8.73 × 10^7^ (Fig. 1a) while CFU g^−1^ of gill samples ranged from 8.67 × 10^3^ to 1.71 × 10^5^ (Fig. 1b). Blood samples plated on MA and BA and incubated at 30 °C ranged from 0 to 28 CFU mL^−1^ and 0 to 19 CFU mL^−1^, respectively (Fig. 1c). All blood samples were culture-positive on at least one media after 7 d of incubation. Of all the isolates counted from blood, 43.8 % were present after 2 d, suggesting a majority of growth occurred following the 2 d mark. A majority of isolates from gill and feces samples were present after 2 d (87.3 and 82.3 %, respectively). There were no significant differences in CFUs between growth media, incubation temperatures, or incubation times within a sample type.

### Sequencing

**Fig. 2:**
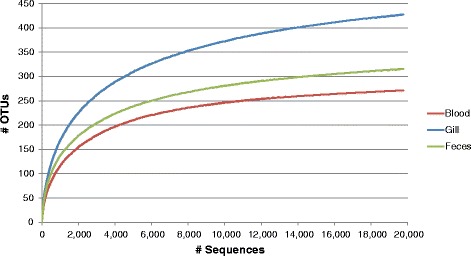
Rarefaction curves for each sample type. Good’s coverage indicated >98 % coverage for all sample types

Sequencing was successful for 19 of 30 samples including: six feces samples, nine gill samples, and four blood samples (Table 2). Inhibitor removal failed to improve sequencing efforts. Number of sequences from individual samples ranged from 950 to 11,888 with 543 total OTUs. Following random sequence selection to standardize sampling effort across samples, number of OTUs decreased to 453. Good’s coverage indicated >98 % sample coverage across all samples (Fig. 2). Sample types differed in terms of expected OTUs (F_2,16_ = 4.02, *p* = 0.038) with feces having a significantly higher number than blood samples, indicating higher bacterial species richness. Shannon evenness indices were not statistically different between sample types.

**Table 2:** Results of 454 pyrosequencing from each Red Snapper individual and sample type

Fish ID	Sample type	Original # sequences	Original # OTUs	Final # sequences	Final # OTUs	Expected # OTUs	Good's coverage	Shannon evennness index
01B	Blood	2263	48	950	43	56	0.991	0.724
04B	Blood	3019	83	950	64	103	0.98	0.735
08B	Blood	950	49	950	49	99	0.991	0.712
10B	Blood	2495	52	950	46	62	0.988	0.665
01G	Gill	3055	74	950	59	103	0.984	0.599
02G	Gill	2581	80	950	68	104	0.981	0.602
03G	Gill	9276	91	950	51	102	0.984	0.533
04G	Gill	3045	81	950	61	101	0.982	0.455
05G	Gill	11888	153	950	90	245	0.963	0.689
06G	Gill	4000	134	950	90	170	0.973	0.762
07G	Gill	4170	109	950	75	247	0.983	0.816
08G	Gill	3140	77	950	64	107	0.986	0.706
09G	Gill	4355	76	950	54	121	0.986	0.649
05 F	Feces	3859	109	950	74	147	0.973	0.545
06 F	Feces	3669	141	950	99	195	0.967	0.704
07 F	Feces	4156	92	950	66	141	0.987	0.8
08 F	Feces	4626	116	950	72	354	0.966	0.512
09 F	Feces	2595	66	950	53	126	0.986	0.727
10 F	Feces	3154	107	950	80	251	0.968	0.591
Average	Blood	2182	58	950	51	80	0.988	0.709
	Gill	5057	97	950	68	144	0.98	0.646
	Feces	3677	105	950	74	202	0.975	0.647

Averages for each sample type are included. Original sequences and # OTUs are from non-standardized data whereas final sequences and # OTUs are after standardizing to 950 sequences per sample (sample 08B)

**Fig. 3:**
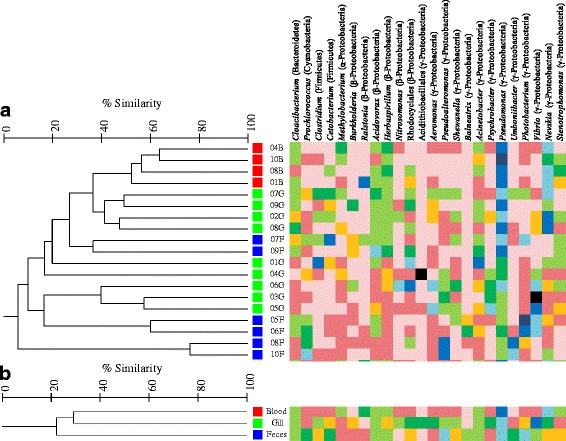
Cluster analysis. Dendrogram is based on percent similarity in OTU abundances for individual samples (tree **a**) and sample type (tree **b**). *Red*, blood; *green*, gill; *blue*, feces. Genus level composition is included for each sample and sample type with genera accounting for 5 % of sequences in at least one sample represented

ANOSIM analysis of OTU abundances indicated significant differences in the bacterial communities between the three sample types (Table 3) with relatively high overlap. Blood microbiota was not significantly different from gill microbiota and these sample types shared 23.9 % of OTUs. Blood and feces samples were statistically separated as indicated by the relatively high *R* value (*R* = 0.444) and low number of shared OTUs (13.5 %). As indicated by the slightly lower *R* value between gill and feces samples (*R* = 0.364), the microbiota of these sample types shared a slightly higher number of OTUs (16.8 %), but they were still significantly different from one another. The four blood samples clustered together with a similarity of 55.2 %, while individuals showed much lower similarity within gill and feces samples (20 %). Feces and gill samples were mixed together in cluster analysis (Fig. 3). Within the total OTUs for each sample type (271, 458, and 321 for blood, gill, and feces, respectively), only 14.8 % of blood OTUs were present in all individuals, and less than 5 % for gill and feces samples. Therefore, there was a high level of variability between the microbiota of individuals within each sample type.

**Table 3:** Analysis of similarities (ANOSIM)

ANOSIM	*p* value	*R* value	Shared OTUs
Global Test	0.014	0.282	68
Blood *vs* gill	0.171	-	174
Blood *vs* feces	0.019	0.444	80
Gill *vs* feces	0.004	0.364	131

Results were calculated using Primer software and corresponding shared OTUs shown were calculated using Mothur

**Fig. 4:**
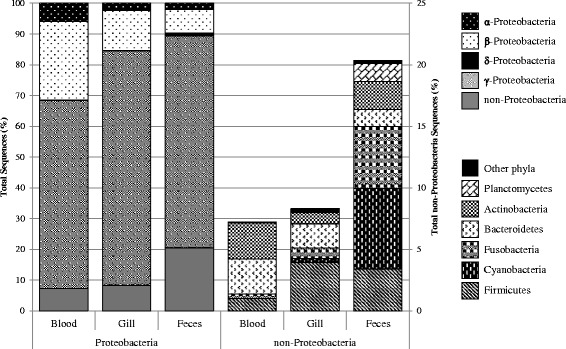
Phylum level composition of each sample type. *Left*, Proteobacteria included; *right*, excluding Proteobacteria. Only phyla accounting for at least 0.1 % of sequences are included. All other phyla are grouped into “Other phyla”

Phylum level analysis of the microbiota (Fig. 4) indicated that Proteobacteria dominated all sample types, specifically the Gammaproteobacteria class. Feces samples contained a larger abundance of non-Proteobacteria including relatively high abundances of Cyanobacteria, Fusobacteria, and Planctomycetes as compared to the other sample types. A majority of the sequences identified from blood that were not Proteobacteria were identified as either Actinobacteria or Bacteroidetes at an abundance of 3 % total sequences each. These two phyla were also present in gill samples, but at lower abundances (1 and 2 %, respectively). Approximately 4 and 3.4 % of the sequences from gill and feces samples, respectively, were identified as Firmicutes as compared to 1 % from blood samples. Less abundant (<0.2 % of sequences) phyla included: Gemmatimonadetes, ws3, and Tenericutes in gill; tm6 and Deinococcus in feces; Nitrospirae and Verrucomicrobia in blood and gill; and tm7 and Spirochaetes in gill and feces.

Dominant genera (at least 5 % of sequences in at least one sample, Fig. 3) indicated high variability in genera abundances between individuals and sample types. In general, blood samples were predominantly *Pseudomonas* and *Nevskia* with larger abundances of *Methylobacterium* and *Stenotrophomonas* as compared to other sample types. Microbiota of samples 03G and 04G were highly dominated by *Vibrio* (81 %) and Acidithiobacillales (76 %), respectively. Although *Vibrio* sequences were identified in a majority of samples, Acidithiobacillales was absent from all other samples but one (05G, < 0.5 % sequences). *Vibrio* was the most common genus present in gill samples. Gill samples also contained a higher abundance of Rhodocyclales, *Clostridium*, *Burkholderia*, *Nitrosomonas*, *Aeromonas*, *Shewanella* and *Psychrobacter* than blood and feces samples. The outgroup formed by samples 08 and 10 F was in part due to high abundances of *Pseudoalteromonas*, *Umboniibacter*, and *Prochlorococcus* as compared to other samples and these numbers contributed to the high abundances of these genera overall in feces samples. The genus *Balneatrix* was only identified in feces samples. Overall feces had higher abundances of *Prochlorococcus*, *Cetobacterium*, and *Photobacterium* as compared to other sample types. Genera shared by all individuals across all sample types include *Pseudomonas*, *Acidovorax*, and *Herbaspirillum. Cloacibacterium*, *Acinetobacter*, and *Nevskia* were also present in a majority of samples.

## Discussion

Increased bacterial loads can cause immune stress in fish, potentially leading to invasion by environmental bacteria [32]. Knowledge on the natural bacterial abundances of fish tissues that are primary entry routes for pathogens is therefore an important aspect of fish health. Red Snapper had similar bacterial loads in tissues susceptible to invasion as other marine fish species, including other species of snappers (genus *Lutjanus*). Feces aerobic heterotrophic counts (average after 7 d = 3.08 × 10^7^ CFU g^−1^) were similar to those seen in other studies on fish gut microbiota [33] and wild marine fish species including Atlantic Cod *Gadus morhua* [34], Daisy Parrotfish *Chlorurus sordidus*, Whitecheek Surgeonfish *Acanthurus nigricans* and Two-Spot Red Snapper *Lutjanus bohar* [35]. Aerobic counts from gill (average 3.59 × 10^4^) were also within normal range for fishes [33], including Atlantic Mackerel *Scomber scombrus* [36], African Red Snapper *L. agennes* [37], and Blackspot Snapper *L. ehrenbergi* [38]. Previous studies reporting bacterial isolation from fish blood did not report aerobic counts. Therefore, only composition of microbiota can be compared with these studies, not abundance of bacteria.

Only 19 of 30 samples were successfully sequenced using this study’s methods. Bile salts [39] and complex polysaccharides [40] present in feces and hemoglobin [41] in blood are known to inhibit PCR reactions; thus, the loss of these samples may be due to the presence of PCR inhibitors, although inhibitor removal did not increase success. In blood samples, small sample size (15 μL per extraction) for DNA extraction may not be sufficient to detect bacteria present in small abundances. Furthermore, the presence of large amounts of host DNA may interfere with bacterial DNA amplification. The number of studies that have detected bacteria in the blood and internal organs of apparently healthy fish [16–20, 22–24, 33, 42, 43] suggest a need for a DNA extraction method optimized for extraction of bacterial DNA from fish blood.

Individual variability was highest in feces samples, with differences of up to 97 % in bacterial community structure. These differences could not be attributed to environmental conditions alone as replicates within the same sample type rarely clustered by site (5 and 6 F only). These differences may be attributable to diet [44], although this was not examined in this study. Host genetics are also known to play a role in shaping microbiota structure [45, 46] and as a result, high variability between individuals is not uncommon in fish microbiota studies [46–49].

The phyla present in Red Snapper gut microbiota were consistent with those reported for other fish species (for a meta-analysis, see Sullam et al. [44]) including other studies on snappers [24, 37, 50, 51] with a community dominated by Proteobacteria, specifically Gammaproteobacteria, and minor phyla including Fusobacteria, Firmicutes, Actinobacteria, Bacteroidetes, and Planctomycetes. Red Snapper had a relatively high abundance of Cyanobacteria (6.6 % of sequences). This phylum is present in the gut of a number of marine fish species [35, 52, 53] including Mangrove Red Snapper *​L. argentimaculatus* [51] but its presence is believed to be due to ingestion of food [54] or extraction of chloroplast DNA [52]. Abundant genera previously reported in association with the gut of marine fish include *Pseudoalteromonas* [55–58], *Cetobacterium* [52, 59], and *Photobacterium* [35, 60–62]. Two individuals had relatively high abundances of *Umboniibacter*, *Pseudoalteromonas*, and *Prochlorococcus*, and these two were highly separated from the rest of the feces samples. These individuals were caught on the same day but at two different sites. As these two sites were nearly identical in terms of environmental conditions, this separation may be due to dietary or genetic differences. A majority of sequences from Mangrove Red Snapper identified as *Vibrio* [51], whereas sequences from Emperor Red Snapper *​L. sebae* were *Vibrio*, *Stenotrophomonas*, and *Photobacterium* [50]. All of these genera were present in Red Snapper feces with *Photobacterium* being the most abundant. Sequences in this genera were primarily identified as *Photobacterium damselae. P. damselae* is a known fish pathogen [63] with high adhesion capability to fish intestinal cells [64], and its presence in apparently healthy fish supports previous reports of *P. damselae* as an opportunistic pathogen [65].

Gammaproteobacteria are often identified as a main component of the fish gill microbiota [66–68], accompanied by Firmicutes [68, 69], Actinobacteria [66, 68] and Bacteroidetes [67]. Many of the common genera identified in Red Snapper gill samples are similar to those of other fish species including *Acinetobacter*, *Aeromonas*, *Psychrobacter*, *Photobacterium*, *Pseudomonas* and *Vibrio* [21, 36, 66–70], providing support for these genera being common members of the fish gill microbiota. Some genera reported in the gill of African Red Snapper *L. agennes* were also found in this study including *Staphylococcus*, *Bacillus*, *Alcaligenes*, and *Escherichia* [37], but on average all these genera represented less than 1 % of sequences in Red Snapper. Other genera not found in Red Snapper included *Klebsiella*, *Proteus*, and *Micrococcus*, supporting previous evidence that fish share some members of the microbiota, while other members are species-specific [71]. Relatively high abundances of unidentified sequences from the orders Acidithiobacillales and Rhodocyclales were present in gill samples. These sequences shared 85–90 % homology with known sequences, and may represent new genera or species within these orders. Members of Acidithiobacillales are rarely reported from fish [72] and presence and abundance of Rhodocyclales seems to vary based on time of year and location [13, 47, 72, 73]. Thus members of these orders may be transient members of the gill microbiota of fishes. Large abundances of *Nevskia* were mainly attributable to sequences closely related to *Nevskia ramosa* and were present in blood samples as well. To our knowledge, in fish, this genus has only been reported from the skin community of brook charr [13, 46].

All presumably healthy individuals sampled in this study displayed positive blood culture growth after 7 d. The high percentage of culture-positive individuals may be a result of a larger sample volume and longer incubation time as compared to previous studies. Cultures in this study were made from 2 mL samples whereas a majority of studies used 10–100 μL [19, 20, 24]. In this study, over 50 % of the isolates grew after 2 d. Similarly, Mylniczenko et al. [23] determined most growth in elasmobranch blood samples occurred after 72 h. Studies on the blood and internal organs of freshwater bony fish stopped incubation after 5 d at most [16, 17, 19, 20], whereas previous studies on marine fish stopped incubation after 2–3 d [22, 24]. Low sample size (ten individuals) may also have influenced our results, as other studies on marine fish have seen positive blood cultures in 25–52 % of fishes with much larger sampling efforts [22–24, 26]. It is important to note that a majority of the bacterial genera identified in the blood of wild Red Snapper have previously been reported as contaminants [74–78] and the high similarity between blood and gill samples may indicate contamination from skin-associated bacteria. Further, fish were caught using rod and reel and skin bacteria could potentially enter the bloodstream through the hook wound. Future studies should investigate bacteremia using appropriate negative controls to rule out contamination.

Many similarities exist between the microbiota found in the blood and internal organs of apparently healthy fish species across studies. All studies that characterized isolates to the genus level in both marine and freshwater species reported members of the genus *Pseudomonas* [18–20, 22–24, 26, 72]. Members of this genus 1) may be permanent residents of the blood microbiota, 2) may be better equipped to penetrate the epithelium of the fish to enter the bloodstream, or 3) may indicate sample contamination. The presence of *Pseudomonas* in multiple fish species across studies makes this genus an interesting target for future investigations. Other genera that are commonly identified in the blood and internal organs of fish include *Achromobacter*, *Aeromonas*, *Bacillus*, *Enterobacter*, *Micrococcus*, *Photobacterium*, *Streptococcus*, *Staphylococcus*, *Stenotrophomonas*, and *Vibrio* [18–20, 22–26, 72]. All of these genera, with the exception of *Achromobacter* and *Stenotrophomonas*, contain species that have been identified as fish pathogens [15]. In this study, sequences from all of these genera were identified in apparently healthy Red Snapper blood with the exception of *Streptococcus*. It is possible that fish blood contains a wide diversity of bacteria and/or bacterial DNA that cannot be detected using culture-based techniques. However as previously mentioned, many of these genera are also commonly reported as contaminants in sequencing-based studies. It is interesting that many of the same bacterial genera have been isolated from a wide variety of fish species using culture-dependent techniques. As this is the first study to use sequencing to survey bacteria in marine fish blood, more studies should be done to determine the true nature of these bacteria.

## Conclusions

This study provides the first characterization of feces, gill, and blood microbiota of Red Snapper from the Gulf of Mexico via pyrosequencing. High individual variability was detected in the gut, gill, and blood microbiota, but all sample types were dominated by Gammaproteobacteria. Many of the identified genera have been isolated from other fish species, including a number of opportunistic bacterial pathogens. Red Snapper is a desirable candidate for aquaculture and knowledge of the natural microbiota structure is essential for understanding the health and disease susceptibility of these fish in captivity. A healthy microbiota provides protection against opportunistic pathogens and this study describes these communities on tissues that are known to be primary entry routes for pathogens. Its role in fish health highlights the importance of understanding microbiota composition for future work on disease prevention using microbial manipulation.

## Methods

### Sample collection

Red Snapper were collected from different sites on different dates to account for variations in bacterial diversity due to geographical location and environmental conditions [71]. A total of ten individuals were collected and sampled to account for inter-individual microbiota variability [46, 48, 49]. Five artificial reef sites (Table 1) were sampled for Red Snapper in the fall of 2013 approximately 15–30 km south of Orange Beach, AL and Pensacola, FL. Hydrographic parameters (depth (m), salinity (psu), temperature (°C), dissolved oxygen (mg L^−1^), fluorescence (mg (m^3^)^−1^) and turbidity (% saturation)) were measured at each site using a Seabird 19*plus* V2 SeaCAT Profiler CTD (Sea-Bird Electronics, Inc., Bellevue, Washington, USA). Two Red Snapper were caught from each site on hook and line using cut squid as bait. Fish were measured (total length, mm) and weighed (kg). Fish were killed by pithing and a muscle sample was removed from one side with a sterilized filet knife. Exposed muscle tissue was dried and sprayed with 70 % ethanol to prevent external contamination and 2 mL of blood was taken from the caudal vein using a sterile needle and syringe. Triplicate samples of 15 μL were placed into sterile microcentrifuge tubes and the remaining sample was preserved on ice for aerobic heterotrophic counts. Total plate counts were performed on marine agar (all sample types) and blood agar (blood samples only) in order to determine total bacterial load of primary pathogen entry routes. Following blood extraction, the outer surface of the operculum was dried and cleaned using 70 % ethanol. The operculum was pulled back to reveal the gill arches and the anterior gill arch was removed using aseptic techniques. To obtain intestinal contents, the ventral surface of the fish was cleaned with 70 % ethanol and opened to reveal the intestine. The lower third of the intestine was removed using clamps to prevent release of fecal material. Feces were extracted and placed into a sterile centrifuge tube. All samples were kept on ice until arrival at the laboratory (approximately 6 h). Samples were labeled according to individual (01–10) and sample type (B = blood, G = gill, F = feces).

### DNA extraction and sequencing

Upon arrival at the laboratory, triplicate 25 mg samples were taken from each gill clip and feces sample. Triplicate gill, feces, and blood samples were taken from each individual to obtain maximum bacterial diversity. Samples were then subjected to DNA extraction with the DNeasy Blood & Tissue Kit (Qiagen, Valencia, CA) according to manufacturer instructions, including pretreatment for Gram-positive bacteria at 37 °C overnight (15 h), proteinase K digestion for one hour, and digestion of RNA using RNase A. DNA was quantified using a spectrophotometer and triplicates were combined in equimolecular amounts to obtain one sample for each sample type from each fish. Roche titanium 454 sequencing was performed using barcoding and primer 27 F (5′-AGRGTTTGATCMTGGCTCAG-3′) to amplify the variable V1-V3 region of the 16S rRNA gene. PCR conditions included an initial denaturation at 94 °C for 3 min followed by 30 cycles of 94 °C for 30 s, 53 °C for 40 s, and 72 °C for 1 min, concluded with a final elongation at 72 °C for 5 min. Sequences were processed using an exclusive analysis pipeline (MR DNA, Shallowater, TX). Barcodes and primers, short sequences (<200 bp), and sequences with a base call error rate of less than 0.3 % (Q < 25), ambiguous base calls, and long (>6 bp) stretches of identical bases were removed. Following denoising and chimera and singleton sequence removal, operational taxonomic units (OTUs) were defined and identified using BLASTn against the Greengenes database [79] at <3 % sequence agreement according to the current accepted prokaryotic species concept [80]. Rarefaction curves, diversity indices (number of OTUs, number of predicted OTUs using the catchall command, Good’s coverage, and Shannon evenness index), and shared OTUs were calculated using Mothur v.1.33.3 [81].

### Aerobic heterotrophic counts

Remaining blood, gill, and feces samples were weighed and diluted 1:1 with sterile phosphate buffered saline (PBS). After homogenization, subsequent 1/10 dilutions were made and plated in six replicates onto Marine Agar 2216 (MA; Difco Laboratory, Detroit, Michigan, USA) and 5 % sheep blood agar (BA; Hardy Diagnostics, Santa Maria, CA). Three of each plate were incubated at 18 and 30 °C for 1 week. Colony forming units (CFUs) were counted after 2 and 7 days.

### Data analysis

Resulting DNA sequences were randomly selected from each sample in order to standardize sampling effort to that of the sample that returned the least number of sequences (950 sequences, sample 08B). Following standardization, ANOVAs were run on number of expected OTUs and Shannon evenness index to determine differences among sample types. Original sequence data in the form of OTU tables was uploaded in Primer v6 (Primer-E Ltd, Plymouth, UK). After standardization (transforming raw OTU abundances to percentages), cluster analysis was used to visualize similarities between samples and analysis of similarities (ANOSIM) was performed between sample types (blood, gill, feces). A genera percent abundance table was loaded into Primer for similarity percentages (SIMPER) analysis to determine the genera responsible for differences between sample types.

## References

[1] FAO (Food and Agriculture Organization of the United Nations) (2014). The State of World Fisheries and Aquaculture 2014.

[2] The World Bank (2013). Fish to 2030 Prospects for Fisheries and Aquaculture.

[3] Bondad-Reantaso MG, Subasighe RP, Arthur JR, Ogawa K, Chinabut S, Adlard R (2005). Disease and health management in Asian aquaculture. Vet Parasitol.

[4] Sihag RC, Sharma P (2012). Probiotics: the new ecofriendly alternative measures of disease control for sustainable aquaculture. Can J Fish Aquat Sci.

[5] Subasighe RP, Bondad-Reantaso MG, McGladdery SE, Subasighe RP, Bueno P, Phillips MJ, Hough C, McGladdery SE, Arthur JR (2001). Aquaculture development, health and wealth. Aquaculture in the Third Millennium.

[6] Pérez T, Balcázar JL, Ruiz-Zarzuela I, Halaihel N, Vendrell D, de Blas I (2010). Host-microbiota interactions within the fish intestinal ecosystem. Mucosal Immunol.

[7] Llewellyn MS, Boutin S, Hoseinifar SH, Derome N (2014). Teleost microbiomes: the state of the art in their characterization, manipulation and importance in aquaculture and fisheries. Front Microbiol.

[8] Verschuere L, Rombaut G, Sorgeloos P, Verstraete W (2000). Probiotic bacteria as biological control agents in aquaculture. Microbiol Mol Biol Rev.

[9] Defoirdt T, Boon N, Sorgeloos P, Verstraete W, Bossier P (2007). Alternatives to antibiotics to control bacterial infections: luminescent vibriosis in aquaculture as an example. Trends Biotechnol.

[10] Evelyn TPT, Iwama G, Nakanishi T (1997). Infection and disease. The fish immune system: organism, pathogen, and environment.

[11] Ringo E, Myklebust R, Mayhew TM, Olsen RE (2007). Bacterial translocation and pathogenesis in the digestive tract of larvae and fry. Aquaculture.

[12] Cipriano R, Cipriano R, Schelkunov I (2011). Far from superficial: microbial diversity associated with the dermal mucus of fish. Health and diseases of aquatic organisms: bilateril perspectives.

[13] Boutin S, Bernatchez L, Audet C, Derom̂e N (2013). Network analysis highlights complex interactions between pathogen, host and commensal microbiota. PLoS Biol.

[14] Noga EJ (2010). Fish Disease: Diagnosis and Treatment.

[15] Austin B, Austin D (2007). Bacterial Fish Pathogens: Diseases of Farmed and Wild Fish.

[16] Proctor BE, Nickerson JTR (1935). An investigation of the sterility of fish tissues. J Bacteriol.

[17] Bisset KA (1948). Natural antibodies in the blood serum of fresh-water fish. J Hyg (Lond).

[18] Evelyn TPT, McDermott LA (1961). Bacteriological studies of fresh-water fish. Can J Microbiol.

[19] Allen N, Pelczar JMJ (1967). Bacteriological studies on the white perch, *Roccus americanus*. Chesap Sci.

[20] Bullock GL, Snieszko SF (1969). Bacteria in blood and kidney of apparently healthy hatchery trout. T Am Fish Soc.

[21] Nieto TP, Toranzo AE, Barja JL (1984). Comparison between the bacterial flora associated with fingerling rainbow trout cultured in two different hatcheries in the north-west of Spain. Aquaculture.

[22] Toranzo AE, Novoa B, Romalde JL, Nunez S, Devesa S, Marino E (1993). Microflora associated with healthy and diseased turbot (*Scophthalmus maximus*) from three farms in northwest Spain. Aquaculture.

[23] Mylniczenko ND, Harris B, Wilborn RE (2007). Blood culture results from healthy captive and free-ranging Elasmobranchs. J Aquat Anim Health.

[24] Arias CR, Koenders K, Larsen AM (2013). Predominant bacteria associated with red snapper from the northern Gulf of Mexico. J Aquat Anim Health.

[25] Tao Z, Bullard SA, Arias CR (2014). Diversity of bacteria cultured from the blood of lesser electric rays caught in the northern Gulf of Mexico. J Aquat Anim Health.

[26] Sevellec M, Pavey SA, Boutin S, Filteau M, Derome N, Bernatchez L (2014). Microbiome investigation in the ecological speciation context of lake whitefish (*Coregonus clupeaformis*) using next-generation sequencing. J Evolution Biol.

[27] National Marine Fisheries Service (2014). Fisheries Economics of the United States 2012.

[28] Cowan JH, Grimes CB, Patterson WF, Walter CJ, Jones AC, Lindberg WJ (2011). Red Snapper management in the Gulf of Mexico: science- or faith-based?. Rev Fish Biol Fisheries.

[29] Saillant EA, Leclercq E, Bardon-Albaret A, Sarkisian B, Apeitos A, Brown-Peterson NJ, et al. Development of aquaculture of the Red Snapper *Lutjanus campechanus*: research on larval nutrition. Proceedings of the Sixty Fifth Annual Gulf and Caribbean Fisheries Institute. 2012;65:352-6.

[30] Amann RI, Ludwig W, Schleifer KH (1995). Phylogenetic identification and in situ detection of individual microbial cells without cultivation. Microbiol Mol Biol Rev.

[31] Ghanbari M, Kneifel W, Domig KJ (2015). A new view of the fish gut microbiome: advances from next-generation sequencing. Aquaculture.

[32] Cahill MM (1990). Bacterial-flora of fishes - a review. Microb Ecol.

[33] Austin B (2006). The bacterial microflora of fish, revised. Scientific World J.

[34] Ringo E, Sperstad S, Myklebust R, Refstie S, Krogdahl A (2006). Characterisation of the microbiota associated with intestine of Atlantic cod (*Gadus morhua* L.) - the effect of fish meal, standard soybean meal and a bioprocessed soybean meal. Aquaculture.

[35] Smriga S, Sandin SA, Azam F (2010). Abundance, diversity, and activity of microbial assemblages associated with coral reef fish guts and feces. FEMS Microbiol Ecol.

[36] Svanevik CS, Lunestad BT (2011). Characterisation of the microbiota of Atlantic mackerel (*Scomber scombrus*). Int J Food Microbiol.

[37] Akinyemi A, Buoro O (2011). Occurrence of bacteria found in gills, skin, buccal cavity of *Lutjanus agennes*, *Pseudotolithus elongatus* and *Sphyraena barracuda* from Lagos Lagoon, Nigeria. J Fish Aquat Sci.

[38] Al-Bahry SN, Mahmoud IY, Al-Belushi KIA, Elshafie AE, Al-Harthy A, Bakheit CK (2009). Coastal sewage discharge and its impact on fish with reference to antibiotic resistant enteric bacteria and enteric pathogens as bio-indicators of pollution. Chemosphere.

[39] Lantz PG, Matsson M, Wadstrom T, Radstrom P (1997). Removal of PCR inhibitors from human faecal samples through the use of an aqueous two-phase system for sample preparation prior to PCR. J Microbiol Methods.

[40] Monteiro L, Bonnemaison D, Vekris A, Petry KG, Bonnet J, Vidal R (1997). Complex polysaccharides as PCR inhibitors in feces: *Helicobacter pylori* model. J Clin Microbiol.

[41] Al-Soud WA, Radstrom P (2001). Purification and characterization of PCR-inhibitory components in blood cells. J Clin Microbiol.

[42] Valdenegro-Vega V, Naeem S, Carson J, Bowman JP, del Real JL T, Nowak B (2013). Culturable microbiota of ranched southern bluefin tuna (*Thunnus maccoyii* Castelnau). J Appl Microbiol.

[43] Yang G, Bao B, Peatman E, Li H, Huang L, Ren D (2007). Analysis of the composition of the bacterial community in puffer fish *Takifugu obscurus*. Aquaculture.

[44] Sullam KE, Essinger SD, Lozupone CA, O’Connor MP, Rosen GL, Knight R (2012). Environmental and ecological factors that shape the gut bacterial communities of fish: a meta-analysis. Mol Ecol.

[45] Smith CCR, Snowberg LK, Caporaso GJ, Knight R, Bolnick DI (2015). Dietary input of microbes and host genetic variation shape among-population differences in stickleback gut microbiota. ISME J.

[46] Boutin S, Sauvage C, Bernatchez L, Audet C, Derome N (2014). Inter individual variations of the fish skin microbiota: Host genetics basis of mutualism?. PLoS Biol.

[47] Larsen AM, Bullard SA, Womble M, Arias CR (2015). Community structure of skin microbiome of Gulf killifish, *Fundulus grandis*, is driven by seasonality and not exposure to oiled sediments in a Louisiana salt marsh. Microb Ecol.

[48] Fjellheim AJJ, Playfoot KJ, Skjermo J, Vadstein O (2012). Inter-individual variation in the dominant intestinal microbiota of reared Atlantic cod (*Gadus morhua* L.) larvae. Aquac Res.

[49] Chiarello M, Villéger S, Bouvier C, Bettarel Y, Bouvier T (2015). High diversity of skin-associated bacterial communities of marine fishes is promoted by their high variability among body parts, individuals and species. FEMS Microbiol Ecol.

[50] Zhou Z, Shi P, He S, Liu Y, Huang G, Yao B (2009). Identification of adherent microbiota in the stomach and intestine of emperor red snapper (*Lutjanus sebae* Cuvier) using 16S rDNA-DGGE. Aquac Res.

[51] Feng J-B, Luo P, De Dong J, Hu CQ (2011). Intestinal microbiota of mangrove red snapper (*Lutjanus argentimaculatus* Forsskal, 1775) reared in sea cages. Aquac Res.

[52] Givens CE, Ransom B, Bano N, Hollibaugh JT (2015). Comparison of the gut microbiomes of 12 bony fish and 3 shark species. Mar Ecol Prog Ser.

[53] Xing M, Hou Z, Yuan J, Liu Y, Qu Y, Liu B (2013). Taxonomic and functional metagenomic profiling of gastrointestinal tract microbiome of the farmed adult turbot (*Scophthalmus maximus*). FEMS Microbiol Ecol.

[54] Ye L, Amberg J, Chapman D, Gaikowski M, Liu W-T (2014). Fish gut microbiota analysis differentiates physiology and behavior of invasive Asian carp and indigenous American fish. ISME J.

[55] Verner-Jeffreys DW, Shields RJ, Bricknell IR, Birkbeck TH (2003). Changes in the gut-associated microflora during the development of Atlantic halibut (*Hippoglossus hippoglossus* L.) larvae in three British hatcheries. Aquaculture.

[56] Korsnes K, Nicolaisen O, Skar CK, Nerland AH, Bergh O (2006). Bacteria in the gut of juvenile cod *Gadus morhua* fed live feed enriched with four different commercial diets. ICES J Mar Sci.

[57] Martin-Antonio B, Manchado M, Infante C, Zerolo R, Labella A, Alonso C (2007). Intestinal microbiota variation in Senegalese sole (*Solea senegalensis*) under different feeding regimes. Aquac Res.

[58] Ringo E, Sperstad S, Kraugerud OF, Krogdahl A (2008). Use of 16S rRNA gene sequencing analysis to characterize culturable intestinal bacteria in Atlantic salmon (*Salmo salar*) fed diets with cellulose or non-starch polysaccharides from soy. Aquac Res.

[59] Ransom B (2003). Intestinal microbial community composition of six Actinopterygii fish species in the southeastern United States.

[60] Hovda MB, Lunestad BT, Fontanillas R, Rosnes JT (2007). Molecular characterisation of the intestinal microbiota of farmed Atlantic salmon (*Salmo salar* L.). Aquaculture.

[61] Wilson B, Danilowicz B, Meijer W (2008). The diversity of bacterial communities associated with Atlantic cod *Gadus morhua*. Microb Ecol.

[62] Ward NL, Steven B, Penn K, Methe BA, Detrich WH (2009). Characterization of the intestinal microbiota of two Antarctic notothenioid fish species. Extremophiles.

[63] Andreoni F, Magnani M. Photobacteriosis: prevention and diagnosis. J Immunol Res. 2014;793817. 10.1155/2014/793817 PMC4058529 24982922

[64] Magarinos B, Romalde JL, Noya M, Barja JL, Toranzo AE (1996). Adherence and invasive capacities of the fish pathogen *Pasteurella piscici*da. FEMS Microbiol Lett.

[65] Labella A, Berbel C, Manchado M, Castro D, Borrego JJ. *Photobacterium damselae* subsp. *damselae*, an emerging pathogen affecting new cultured marine fish species in southern Spain. In: Aral F, Dogu Z, editors. Recent Advances in Fish Farms. Croatia: InTech; 2011. p. 135–52.

[66] Al-Harbi AH, Uddin N (2003). Quantitative and qualitative studies on bacterial flora of hybrid tilapia (*Oreochromis niloticus* × *O. aureus*) cultured in earthen ponds in Saudi Arabia. Aquac Res.

[67] Kapetanovic D, Kurtovic B, Teskeredzic E (2005). Differences in bacterial population in rainbow trout (*Oncorhynchus mykiss* Walbum) fry after transfer from incubator to pools. Food Technol Biotechnol.

[68] Latha N, Mohan MR (2013). The bacterial microflora in the fish organs-a public health aspect. Indian J Adv Chem Sci.

[69] Wang WW, Zhou ZG, He SX, Liu YC, Cao YN, Shi PJ (2010). Identification of the adherent microbiota on the gills and skin of poly-cultured gibel carp (*Carassius auratus gibelio*) and bluntnose black bream (*Megalobrama amblycephala* Yih). Aquac Res.

[70] Steinum T, Sjastad K, Falk K, Kvellestad A, Colquhoun DJ (2009). An RT PCR-DGGE survey of gill-associated bacteria in Norwegian seawater-reared *Atlantic salmon* suffering proliferative gill inflammation. Aquaculture.

[71] Larsen A, Tao Z, Bullard SA, Arias CR (2013). Diversity of the skin microbiota of fishes: evidence for host species specificity. FEMS Microbiol Ecol.

[72] Boutin S, Sevellec M, Pavey SA, Bernatchez L, Derome N (2012). A fast, highly sensitive double-nested PCR-based method to screen fish immunobiomes. Mol Ecol Resour.

[73] Zarkasi KZ, Abell GC, Taylor RS, Neuman C, Hatje E, Tamplin ML (2014). Pyrosequencing-based characterization of gastrointestinal bacteria of Atlantic salmon (*Salmo salar* L.) within a commercial mariculture system. J Appl Microbiol.

[74] Salter S, Cox M, Turek E, Calus S, Cookson W, Moffatt M (2014). Reagent and laboratory contamination can critically impact sequence-based microbiome analyses. BMC Biol.

[75] Tanner M, Goebel B, Dojka M, Pace N (1998). Specific ribosomal DNA sequences from diverse environmental settings correlate with experimental contaminants. Appl Environ Microb.

[76] Grahn N, Olofsson M, Ellnebo-Svenlund K, Monstein H, Jonasson J (2003). Identification of mixed bacterial DNA contamination in broad-range PCR amplification of 16S rDNA V1 and V3 cariable regions by pyrosequencing of cloned amplicons. FEMS Microbiol Lett.

[77] Barton H, Taylor N, Lubbers B, Pemberton A (2006). DNA extraction from low-biomass carbonate rock: an improved method with reduced contamination and the low-biomass contaminant database. J Microbiol Methods.

[78] Laurence M, Hatzis C, Brash D (2014). Common contaminants in next-generation sequencing that hinder discovery of low-abundance microbes. PLoS Biol.

[79] DeSantis TZ, Hugenholtz P, Larsen N, Rojas M, Brodie EL, Keller K (2006). Greengenes, a chimera-checked 16S rRNA gene database and workbench compatible with ARB. Appl Environ Microb.

[80] Rossello-Mora R, Amann RI (2001). The species concept for prokaryotes. FEMS Microbiol Rev.

[81] Schloss PD, Westcott SL, Ryabin T, Hall JR, Hartmann M, Hollister EB (2009). Introducing mothur: Open-source, platform-independent, community-supported software for describing and comparing microbial communities. Appl Environ Microb.

